# Dentist perceptions about the value of teledentistry

**DOI:** 10.1186/s12903-022-02208-z

**Published:** 2022-05-13

**Authors:** Tamanna Tiwari, Vuong Diep, Eric Tranby, Madhuli Thakkar-Samtani, Julie Frantsve-Hawley

**Affiliations:** 1 Anschutz Medical Campus School of Dental Medicine, University of Colorado, 13065 East 17th Avenue, Aurora, CO 80045 USA; 2 CareQuest Institute for Oral Health, Boston, MA USA

**Keywords:** Dentist perceptions, Access to care, Insurance

## Abstract

**Background:**

Teledentistry has expanded access to oral health care by allowing patients and providers the option to receive care using technology and telecommunications. This study used a cross-sectional, mixed-methods design to evaluate dentists’ perceptions in the United States and understanding of the value and scope of teledentistry in their practices and to adopt virtual encounters as a care delivery methodology.

**Methods:**

This study used a cross-sectional, mixed-methods design. The DentaQuest Partnership for Oral Health Advancement (now CareQuest Institute for Oral Health) conducted an electronic survey of providers in the DentaQuest Network that assessed the impact of COVID-19 on dental practices' patient volume, staffing, dental insurance carriers, treatment protocols, and the office's pre-and post-COVID finances. A total of 2767 dental providers completed the survey with a response rate of 13%. Qualitative interviews were then conducted with ten providers to get more in-depth information on teledentistry. Descriptive statistics summarize the survey population. Thematic analysis, which allows both deductive and inductive approaches, were used to analyze the interviews.

**Results:**

About 23% of the dentists used teledentistry or virtual platforms. Findings illustrate that early adopter dentists were more likely to perceive the benefits of teledentistry as being more significant than its drawbacks. Late/resistant adopters to teledentistry were less aware of its benefits and were more focused on the drawbacks, such as upfront cost. Late adopters were also concerned about the level of care delivered through teledentistry.

**Conclusions:**

This study explored dentists’ perceptions of teledentistry. Expanding access to care was recognized as one of the greater values of teledentistry.

## Background

"Teledentistry is the practice of delivering oral health care, consultation, and education using information technology and telecommunications" [1]. In the past decade, teledentistry has grown in areas where there is a dental workforce shortage and for rural and underserved populations. The scope of practice of teledentistry includes emergency triage, full consultations, follow-up, and oral health education and promotion. Several forms of teledentistry are used, including synchronous or live visits, asynchronous visits, remote patient monitoring, and mobile health [2–5].

Several studies have evaluated the teledentistry applications' reliability, validity, and efficacy with positive outcomes [5–7]. The majority of the research in these areas reported that teledentistry had similar or better outcomes than the conventional healthcare delivery [5–7]. In addition, studies have reported that teledentistry can be a cost-effective and cost-minimizing method of care for rural areas [8, 9].

During the COVID-19 pandemic, teledentistry aided in preventing the delay and disruption of dental care. Virtual encounters helped patients, especially older and immunocompromised, access dental care without leaving the safety of their homes, reducing their potential exposure [8, 9]. During the pandemic, initial and follow-up, teledentistry visits helped to increase patient compliance and establish a stronger patient-provider relationship [10, 11]. Despite feasibility reports and evidence-based research related to teledentistry, few studies have captured the dentists' perceptions regarding a virtual encounter with the patient, barriers they have faced in bringing this technology to their dental practice, and the success they have experienced using this technology teledentistry. This report presents the findings of an evaluation of dentists’ practicing in the United States to understand the value and scope of teledentistry in their practices and the adoption of virtual encounters as a care delivery methodology. The significance of this study lies in understanding the perspectives of the dentist in using teledentistry, as will be the end-users of this technology that can benefit patient care. This study also highlights the challenges faced by dentists in using this technology and the concerns they may have in using virtual platforms.

## Methods

The study was exempted by the Western Institutional Review Board and fully accredited by the Association for the Accreditation of Human Research Protection Programs. This study used a cross-sectional, mixed-methods design. The DentaQuest Partnership for Oral Health Advancement, now the CareQuest Institute for Oral Health, conducted an electronic survey from August to September 2020 by sending an emailed invitation and link to 21,617 DentaQuest-enrolled dental providers in more than 20 states. Up to three reminders were sent to encourage completion. The survey assessed the impact of COVID-19 on dental practices' patient volume, staffing, dental insurance carriers, treatment protocols, and the office's pre-and post-COVID finances. The survey also included questions regarding the current use of teledentistry or virtual platforms and services provided by teledentistry platforms. Dentist demographic information on age, race, and gender was also collected. A total of 2767 dental providers partially or fully completed the survey for a response rate of 13%.

The qualitative study used a thematic approach and semi-structured, in-depth interviews for the methodology. The research team developed the interview questions to get more in-depth information on teledentistry based on the survey questions. The team used an iterative process to build themes and subsequently generate related questions. The themes were:

- Perceptions about teledentistry.
- The scope of teledentistry.
- Enablers for the adoption of teledentistry.
- Barriers faced in the adoption and practice of teledentistry.

Interview questions:

1. Please describe your perceptions and ideas about using teledentistry in your office?
2. How does teledentistry help in patient care?
3. How did COVID-19 impact the use of teledentistry in your dental office? Can you pls provide a few examples of increased/decreased use of teledentistry?
4. What are your concerns about teledentistry?
5. What barriers have you faced in using teledentistry in your dental office. Can you provide some examples?

The interviewer used several probes to elicit more in-depth information on particular themes, including asking the dentist to elaborate on the answer they provided. Examples of a few probes: give examples of situations where you were able to provide care to your patients using teledentistry; can you give an example of the quality of care concerns you mentioned. The in-depth interviews aimed to understand the perceptions of the dental providers towards teledentistry and enablers and barriers to the acceptance and use of teledentistry.

The sample of DentaQuest enrolled dental providers was recruited from ten different states using the convenience sampling strategy. Ten participants were emailed and the goal of the study was explained. Once they agreed to participate, a video call ink was sent for a choice of their date and time. A total of ten dental providers were interviewed for the qualitative part of the study and the authors had no prior relationship with the participants. Each interview lasted between 30 and 60 min. All ten interviews were conducted using a video platform hosted by two authors (Tiwari T. and Diep V) to improve the reliability and rigor of the research method. All interviews were recorded and transcribed verbatim by a third party. The interview transcriptions were anonymized before data analysis. Apart from the interview data, the survey contained one open-ended question related to barriers faced by using teledentistry. Responses to that question were included in the qualitative analysis apart from the interviews.

### Data analysis

We used descriptive statistics to summarize the survey population by age, gender, race, current telehealth utilization, patient volumes, the proportion of patients covered under managed care contracts or Medicaid, and expectations around long-term changes in dentistry. Further, we ran a multivariable logistic regression model to examine factors associated with current telehealth utilization by dentists.

The first author of this paper completed the qualitative analysis. She has extensive training in qualitative analysis and has published several peer-reviewed manuscripts with qualitative and mixed-methods analysis. This study used thematic analysis, which allows both deductive and inductive approaches to raise the perspectives of the participating dentists to a level of conceptualization of emerging themes. The data generated from the interviews were sufficient in scope to produce themes and codes. Repetition in the ideas and perspectives was seen, and the coder reached saturation within the ten interviews. All analysis was completed in ATLAS.ti (Scientific Software Development GmbH).

## Results

### Quantitative results

Table 1 provides descriptive results for the dentists who participated in the survey. Dentists aged 35–44 comprised the largest portion, 28%, of respondents. Of the individuals who reported their gender, more females than males (43% vs. 28%). About 48% of the participating dentists were White, 14% were Asian, 11% were Hispanic, 7.6% were black, 1% were American Indian, and 0.2% were Native Hawaiian or Pacific Islander. About 65% of the dentists reported that their patient volume declined during the pandemic. About 23% of the dentists were using teledentistry or virtual platforms. About 61% of the dentists said that less than 50% of their patients were covered under a managed-care contract. When asked about the impact on their practices, 93% expected the adoption of teledentistry to lead to a long-term change in how they practice dentistry.

**Table 1:** Descriptive results from the COVID provider survey

	Frequency	Percent
*Age*		
18–34	367	15.96
35–44	647	28.14
45–54	565	24.58
55–64	512	22.27
65 +	208	9.05
*Gender*		
Female	994	43.24
Male	648	28.19
Other	3	0.13
Would rather not say	122	5.31
Missing	532	23.14
*Race/Ethnicity*		
White	872	47.83
Asian	258	14.15
Hispanic	199	10.92
Black	139	7.62
American Indian	18	0.99
Native Hawaiian or Pacific Island	5	0.27
Others/prefer not to say	332	18.21
*Use telehealth or virtual platforms*		
No	1103	65.65
Not currently but in future	187	11.13
Yes	390	23.21
*Patient volume*		
More	286	12.94
Less	1429	64.63
No change in volume	496	22.43
*Proportion of the patients covered under a managed care contract*		
< 50%	1081	61.04
> 50%	186	10.50
Don’t know/prefer not to answer	504	28.46
*Long term changes*		
Expect long term changes in practice	2138	93.00
Don’t expect long term changes in practice	161	7.00

Table 2 provides the result of the logistic regression analysis for using teledentistry. Dentists who had higher volume of patients (OR = 0.52, CI = 0.34, 0.80; p = 0.003) or no change in the volume patients (OR = 0.53, CI = 0.38, 0.74; p = 0.000) due to COVID-19 reported lower odds of using teledentistry. Dentists seeing greater than 50% of Medicaid patients reported higher odds of using teledentistry (OR = 1.35, CI = 1.01, 1.79; p = 0.038). There was no impact of the dentist's gender and race on the use of teledentistry. However, dentists over the age of 65 years had lower odds of using teledentistry (OR = 0.40, CI = 0.22, 0.76; p = 0.005). Dentists who did not expect long-term changes in the practice of dentistry also reported lower odds of using teledentistry (OR = 0.40, CI = 0.22, 0.76; p = 0.005).

**Table 2:** Association between patient variables, demographic variables and long-term change expectation and teledentistry use by dentists

	Odds ratio	95% conf. interval	P value
*Age*	(18–34: Ref)		
35–44	1.33	0.89, 2.02	0.168
45–54	0.91	0.60, 1.39	0.669
55–64	0.66	0.42, 1.02	0.063
65 +	0.40	0.22, 0.76	0.005
*Gender*	(Female: Ref)		
Male	1.17	0.87, 1.53	0.314
*Race/Ethnicity*	(White: Ref)		
Black	0.94	0.57, 1.54	0.807
Hispanic	1.05	0.64, 1.72	0.842
Asian/American Indian/Native Hawaiian/Pacific Islander	1.02	0.69, 1.5	0.915
Other/prefer not to answer	0.59	0.37, 0.93	0.024
*Patient volume*	(less: Ref)		
More volume	0.52	0.34, 0.80	0.003
No change in volume	0.53	0.38, 0.74	0.000
*Proportion of the patient’s covered by Medicaid*	(< 50%: Ref)		
> 50%	1.35	1.01, 1.79	0.038
Prefer not to answer/don’t know			
*Proportion of the patients covered under a managed care contract*	(None: Ref)		
< 50	0.94	0.67, 1.31	0.708
> 50%	0.99	0.63, 1.55	0.982
Prefer not to answer/don’t know	0.76	0.52, 1.01	0.155
*Long term changes*			
Don’t expect long term changes in practice	0.44	0.24, 0.81	0.007

Logistic regression

### Qualitative results

The interviews were conducted with ten dentists, and all of them were in private practice. Six participants were males and four females. Our analysis of the semi-structured interviews revealed that the dentists participating in the interviews belonged to two groups:

1. Early adopters of teledentistry
2. Late adopters.

These two groups were defined based on the awareness, knowledge, and readiness to use teledentistry.

### Early adopters of teledentistry group

**Fig. 1:**
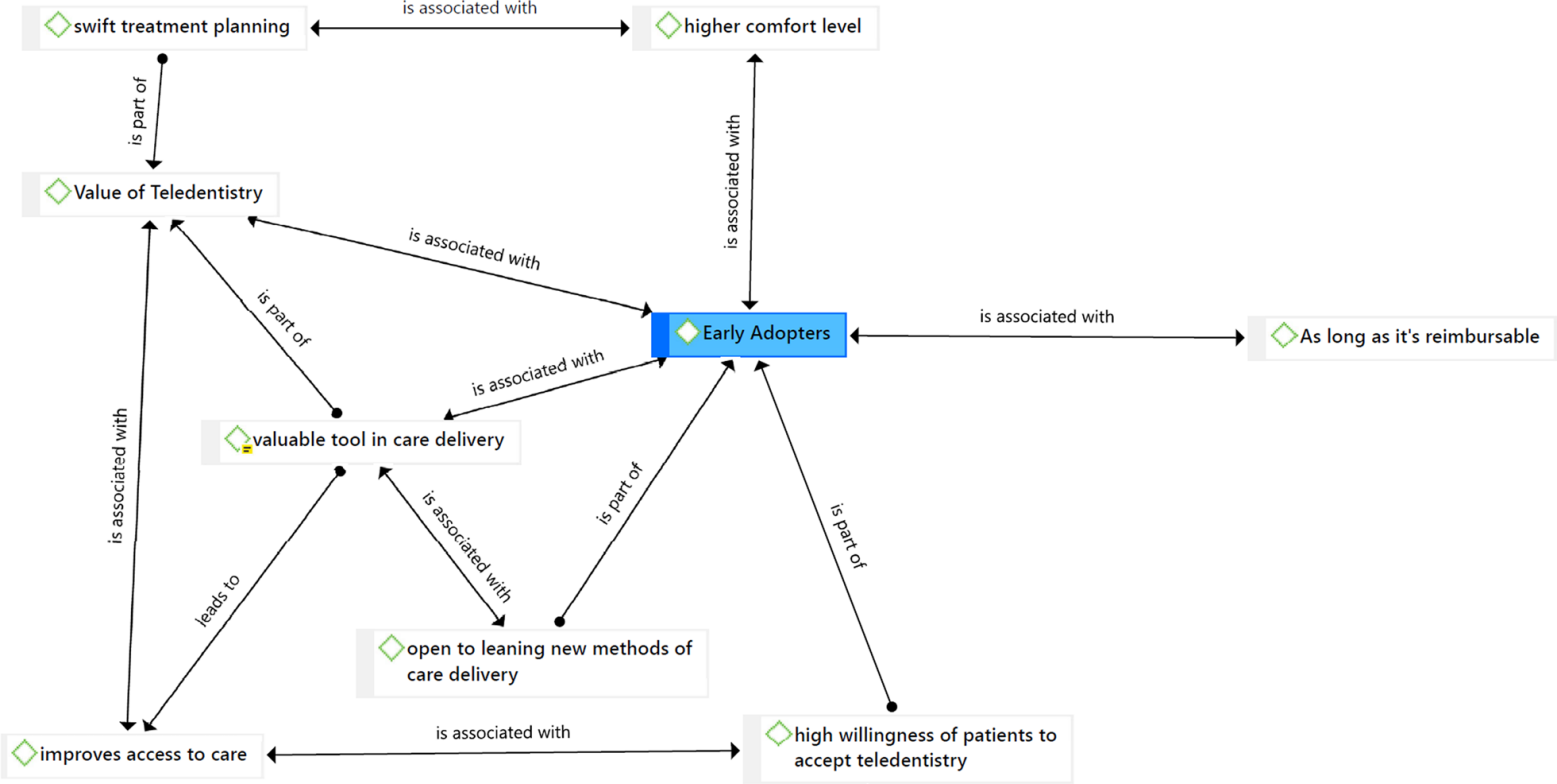
Enabling factors for dentist to adopt teledentistry

Early adopters were dentists already using or open to using teledentistry in their dental office and described the value and advantages of this technology for them and their patients. Dentists in the early adopters’ group discussed the value of teledentistry within their practice and for their patients (Fig. 1). The dentists shared how the components of teledentistry allowed them to provide care for patients during the COVID-19 pandemic. Several dentists in this group shared that they see teledentistry as a valuable tool in care delivery and felt it could have a significant role in the long-term future of dentistry—even post-pandemic. They discussed the future of dentistry in which virtual encounters would have the same standing as an in-person encounter, with dental teams involved in capturing the clinical and radiographic data asynchronously. They also acknowledged that dentists in many states were far from understanding the scope and usefulness of virtual encounters in their practice.

I think it is a valuable tool if it prevents someone from having to come in, especially if they live an hour away.

This is almost the same thing as an in-person encounter because we have many of the same elements that we could capture remotely.

The dentists who were early adopters of teledentistry and considered it a valuable tool in care delivery reached this conclusion due to several underlying factors. They discussed the benefits of teledentistry in swift treatment planning, especially during the pandemic. They said teledentistry allowed them to discuss the patient's chief oral health concerns and conduct a consultation with the patient using the virtual platform. This reduced the patient’s time in the dental chair, which minimized exposure time for both the patient and the dental team. The dentists in the early adopter group acknowledged that reducing the need for chair time could allow them to provide care to more patients.

Dentists in the early adopter group also noted that teledentistry improved access to care for the population they served. Dentists used teledentistry to triage patients, consult on virtual platforms, prescribe medications, and conduct oral health education and promotion. The dentists would receive pictures from patients and use those during the virtual consult and schedule the patients for procedures later. A few dentists said that teledentistry was helping them see patients who lived in rural areas or areas with a shortage of dentists. Some dentists mentioned that they had been using virtual encounters for a few years, but the frequency increased during the pandemic. The early adopters reported that their patients were willing and open to using a virtual platform. In addition, these dentists said their patients were satisfied with the outcomes of the virtual consult and grateful that they did not have to leave the safety of their homes during the pandemic.

I think there is an opportunity that we can try to support people that are geographically separated even among our rural offices that are really out in the country.

A virtual encounter can work for somebody who lives four hours away, and you just want to try to manage them and make sure that they can get in for an appropriate appointment.

I have leveraged virtual dental encounters for a couple of years because I see people from mountain communities that are two and a half, three hours away from my practice.

I thought patients took us up on having a more robust virtual encounter where we spent a lot of time talking about them, their children's oral health, and what they needed to do. It always surprises me how well parents can point their iPhone into their kids' mouths to tell us what's going on.

The early adopter dentists were more comfortable using virtual platforms. In addition, they were open to learning new methodologies of care delivery. Some started with telephone consultations and transitioned to virtual platforms and software as they learned more about the Health Insurance Portability and Accountability Act (HIPPA) regulations and the different platforms available. Several dentists stated that acceptance of virtual encounters could be improved as the technology becomes simpler to set up, easier to use, and demonstrates cost-effectiveness.

We created a customized proprietary teledentistry platform based on teams where we've got a Microsoft platform. We developed that, and that was really helpful. We used that through the shutdown for supporting emergencies.

We will have to reach a certain threshold of dental providers who are used to working in this kind of environment before it becomes commonplace and accepted.

### Late adopters group

**Fig. 2:**
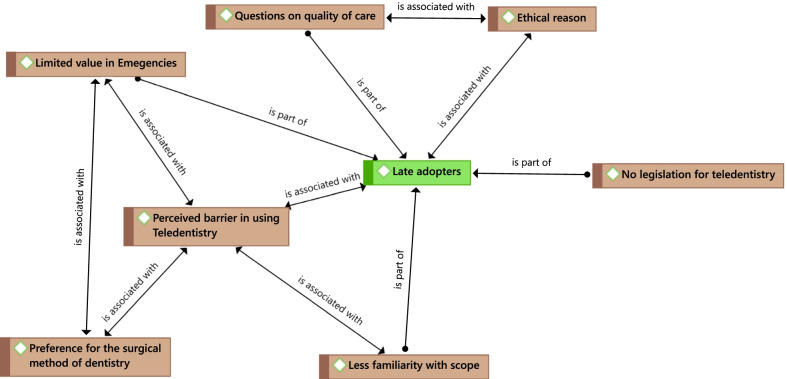
Perceived barriers by dentists in using teledentistry

The dentist in this group were not currently using teledentistry and brought up their concerns and discussed issues they thought were barriers to using teledentistry in their dental practice. Dentists within this group brought up the ethical concerns and quality of care as reasons for not using teledentistry (Fig. 2). A few dentists mentioned that they were not comfortable charging their patients for teledentistry because they were not convinced about the quality of care they could provide through a virtual encounter.

There are a lot of dentists who are very, very averse to the idea of telehealth because there's always this question of quality of care.

We felt that we were trying to sell something that wasn't warranted because the patients had to pay a fee, and we didn't feel comfortable charging them for something that we felt was not useful.

In the late adopter’s group, dentists referred to teledentistry as the trickle-down technology from medicine and how virtual encounters are more useful in medicine than dentistry. They contrasted the hands-on nature of dentistry compared to medicine. They felt virtual encounters had limited value and scope in dentistry. Thus, they were reluctant to invest their time in learning a new methodology for care. They also voiced a preference for the surgical method of dentistry. For this reason, virtual encounters had less relevance to their practices. In addition, several dentists within this group spoke about the limited capacity of the virtual encounter in handling dental emergencies.

That just doesn't apply to dentistry. It's fine for medicine.

You know dentists; our comfort zone is very different from a physician because we can't practice unless we're right in somebody's face.

I think telehealth has a limited value for true emergencies because there**'s** a lot that you gain, in dentistry at least, by radiograph and a clinical examination that would be missed.

Late adopters seemed to be less familiar with the potential benefits that teledentistry could bring to their dental practice and were more reluctant to invest time to learn new technology.

**Fig. 3:**
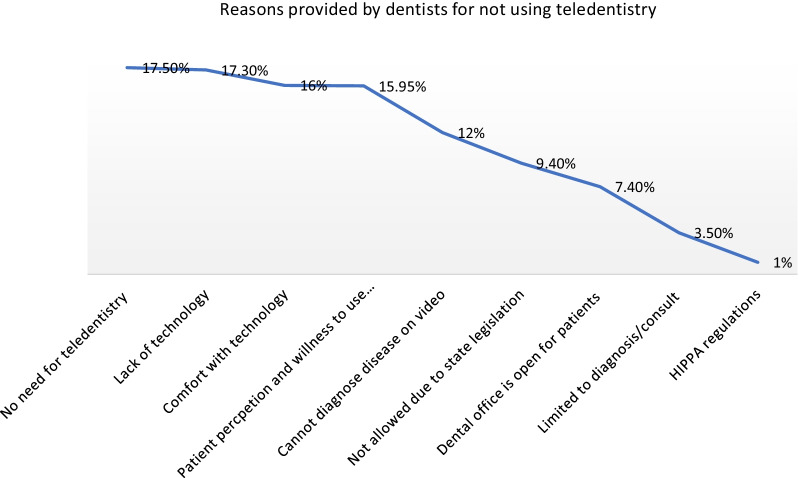
Reasons provided by dentist for not using teledentistry in their practice. *Open-ended responses from the survey

In addition to the interviews, an open-ended survey question asked dentists why they did not use teledentistry, and 41% responded. The primary reason cited in the open-ended questions was similar to that of the interviews. Roughly 1 in 5 dentists (18%) said there was no need for teledentistry in their practice, 17% said that their practice lacked the technology, and 16% said that they were not comfortable using the technology. Only 1% of the dentist said they were not familiar with HIPAA regulations related to virtual encounters (Fig. 3).

Both the early and late adopter groups did mention the role of state legislation in teledentistry and payment by insurers for virtual encounters. All early adopters were practicing in states that allowed legislation for teledentistry, and insurers paid for the virtual encounters. Although, these could be temporary measures that were implemented during the pandemic and may not be sustained for an extended period.

## Discussion

Although teledentistry has been used sparsely for a long time, the pandemic helped to hasten the adoption of this technology-driven care by private dental offices in the U.S., and dentists were able to consult, monitor, and triage patients using teledentistry technology [12, 13].

This study used a mixed-methods approach to understand dentists’ perceptions of teledentistry. This study showed that less than a quarter of the dentists surveyed had used teledentistry, while 11% planned on using it in the future. The study also showed that dentists who were younger or had more than 50% of their patients covered by Medicaid were more likely to report the use of teledentistry. Dentists who did not experience a pandemic-induced change in inpatient volumes and did not expect any long-term change in their dental practice were more likely not to use teledentistry.

Within the qualitative results, we saw a range of perceptions. The early adopters identified the value of teledentistry and adopted it in their practice to improve access to care for their patients and minimize delays in consideration during the pandemic. Expanding access to care is recognized as one of teledentistry’s greater values, which was demonstrated before and during the pandemic [14, 15]. Several universities, foundations, and other stakeholders have implemented different models for teledentistry, providing evidence of improving access to care for underserved, vulnerable, and remote communities [16–19]. Several models have shown that teledentistry is an effective tool in providing access to care for rural communities to general and specialist dental consults and subsequent care [20]. Teledentistry provides access to dental care and reduces travel miles, costs, time, and suffering [8, 9].

The late adopters questioned the quality of care through teledentistry, had ethical concerns about charging their patients for teledentistry, and felt HIPAA regulations might preclude its use in their practices.

Several studies done during the same time have revealed similar results. Dentists’ perceptions about teledentistry services vary from readily accepted, cautiously accepting to non-acceptance of teledentistry [21, 22]. Although studies have reported that the awareness is high among dentists, the use may be less than expected due to challenges faced in gaining skills for new technology use, concerns related to infrastructures, such as internet access, hardware requirement, supply chain issues, and added cost can pose challenges in the use of teledentistry use [23, 24].

Based on this mixed-methods analysis, we reach several recommendations. First, those seeking to encourage the use of teledentistry must recognize that dentists are likely to explore these modalities in a careful, multi-stage sequence that closely resembles the journey being taken by physicians. Research shows that primary care providers had a 15% percent adoption rate for telehealth, which was more significant in rural areas [25]. The barriers perceived by physicians are similar to the perception of the dentists in this study [25, 26]. Our discussion with the early adopters revealed that dental providers are evaluating the benefits of virtual encounters with their patients, and adopting teledentistry would be a phased process, which will involve education, training, and a cost–benefit evaluation of using teledentistry.

Second, the late adopter group is not defiant in their views; indeed, many could become more receptive to teledentistry if their key questions or concerns are addressed. Health system administrators, clinic managers, Medicaid officials, and other leaders in healthcare and dentistry can reduce resistance by bringing clarity. For example, Medicaid and other insurers could issue bulletins or other forms of guidance that clarify the HIPAA compliance in teledentistry. Providing training to practicing dentists and dental students would be one of the other essential steps in increasing the acceptance of teledentistry. Dental schools have started to adopt teledentistry in their curricula, exposing students to the technology, benefits of use, and the helping future dentists be more knowledgeable in this area [27, 28].

Third, federal, state, and insurer regulations are likely to play a critical role in adopting teledentistry. Both groups of dentists agreed that these regulations are pivotal in making modalities of care more or less attractive to providers. Several states have passed emergency rules or legislation to promote the use of teledentistry during the pandemic. Sustaining these measures could be pivotal for the growth of teledentistry [14, 23]. A few public and private insurance carriers have started to reimburse for virtual patient encounters during the pandemic, but this sustainability is in question. Insurers can encourage more patient encounters through live video and store-and-forward mechanisms by covering these forms of teledentistry. Insurers should not place undue restrictions on using the consultation code for teledentistry and should pay a reasonable fee for the code so providers will use it [29]. In addition, states should provide clarity on the issues of HIPAA compliance and liability insurance for providers using teledentistry; some of the applicable laws were written before the use of this technology in dentistry. Bringing clarity and updating laws related to teledentistry could encourage many dentists could get teledentistry into their dental practice model.

Lastly, research in this technology area needs to be ramped up to provide evidence of effectiveness—that will boost the confidence of the dentists who are not using the technology yet. Several new applications were developed to assist in telediagnosis of dental caries and oral cancer using cell phones and video conferencing [30–32]. Some studies have provided evidence that teledentistry diagnosis of dental caries and impacted 3^rd^ molars is reliable [33, 34]. However, we need more research that tests the sensitivity and specificity of these tools compared to traditional oral exams.

As in any research, there are some limitations of this research. We conducted interviews with ten dentists in ten different states, and the qualitative approach included a sample of 2767 from over 20 states; therefore, the sample had a broad reach and covered several states to understand the environments in which dentist practices were situated. However, it is not possible to say that this is a representative sample of dentists; that would require further research with a larger sample**.**

## Conclusion

In conclusion, this study explored dentists’ perceptions of teledentistry in several states in the United States as the pandemic expanded dentistry’s interest in virtual care. This study highlights the cautious optimism of dentists who started using the technology early and the thoughtful dissent of others who have not adopted this technology due to their perceptions of ethical concerns and quality of care issues. These perceptions help to identify potential strategies for easing concerns and developing teaching and training programs and are eventually vital to the uptake and suitability of the use of teledentistry.

## Data Availability

The data used and/or analyzed during the current study are qualitative and are available from the corresponding author upon reasonable request.
